# DNA Barcoding of Wild Plants with Potential Medicinal Properties from Faifa Mountains in Saudi Arabia

**DOI:** 10.3390/genes14020469

**Published:** 2023-02-12

**Authors:** Fatmah Ahmed Safhi, Salha Mesfer Alshamrani, Abdullah Farag Mohammed Bogmaza, Diaa Abd El-Moneim

**Affiliations:** 1 Department of Biology, College of Science, Princess Nourah bint Abdulrahman University, P.O. Box 84428, Riyadh 11671, Saudi Arabia; 2 Department of Biology, College of Science, University of Jeddah, Jeddah 21959, Saudi Arabia; 3 Department of Botany, Faculty of Sciences, Al-Asmarya University, Zliten 218521, Libya; 4 Department of Plant Production (Genetic Branch), Faculty of Environmental Agricultural Sciences, Arish University, El-Arish 45511, Egypt

**Keywords:** wild plants, Arabian Peninsula landscape, folk medicine adulteration, ITS, rbcL

## Abstract

Wild medicinal plants are the main source of active ingredients and provide a continuous natural source for many folk medicinal products, a role that is important for society’s health with an impressive record of utilization. Thus, surveying, conserving, and precisely identifying wild medicinal plants is required. The current study aimed to precisely identify fourteen wild-sourced medicinal plants from southwest Saudi Arabia, within the Fifa mountains area located in Jazan province, using the DNA barcoding technique. Two DNA regions (nuclear ITS and chloroplast rbcL) were sequenced and analyzed for the collected species using BLAST-based and phylogeny-based identification methods. Based on our analysis, ten of the fourteen species were successfully identified by DNA barcoding, five were identified as morphologically inspected, and three were morphologically indifferent. The study was able to distinguish some key medicinal species and highlight the importance of combining morphological observation with DNA barcoding to ensure the precise identification of wild plants, especially if they are medicinally relevant and associated with public health and safety usage.

## 1. Introduction

There is no doubt that medicinal plants are used by a substantial portion of society, and their use is widely recognized. The use of herbs for maintaining human health, especially for chronic diseases, has been practiced worldwide for centuries [1]. In developed countries, people are increasingly using traditional medicine as an alternative to, or alongside, modern medicine [2]. Despite the commercialization of traditional medicine over the last few decades, many medicinal plants remain gathered from the wild [3]. 

It is imperative that medicinal plants are correctly identified if they are to be used in a safe manner [4,5]. The majority of medicinal plants are classified based on morphological characteristics by expert botanists or by the use of analytical techniques to determine their quality (for example, organoleptic, macroscopic, microscopic, and chemical profiling methods) [4,5]. Nevertheless, neither morphological features nor previous methods can easily identify related species, particularly in cases involving powders or processed products obtained from plants [4,5]. In this regard, species adulteration and the use of spurious materials have become increasingly important concerns for health and safety reasons [4,5].

The technique of identifying biological specimens using DNA short sequences is called DNA barcoding [6,7]. A DNA barcode method for global species identification was first developed by Hebert et al. [8], attracting worldwide attention [9,10,11]. Plant DNA barcoding is imperative for the conservation and utilization of plants, as well as to identify species accurately [12]. Aside from identifying species from materials such as roots, seeds, and pollen, this technique can also be used to identify plant mixtures from air, soil, or water samples [13]. DNA barcoding has been widely recognized as an effective and practicable technique among different medicinal plants (e.g., [4,6,14,15,16,17,18]). As mentioned above, DNA barcoding can resolve the difficulties associated with species identification. In order to detect new species and authenticate known species, the DNA barcoding method uses sequence differences in short and standardized DNA regions [8,19,20]. The use of DNA barcoding for species authentication could improve the work and effectiveness of specialists, as well as make the process of authentication more accessible to non-specialists [11,21,22]. Plant taxonomic studies are increasingly using DNA barcode data as additional molecular evidence. The trend of combining morphological, chemical, and genetic markers for species identification has become increasingly important, with DNA barcoding becoming one of the most efficient methods for identifying medicinal plant species [23].

In Saudi Arabia, the flora is one of the richest areas of biodiversity in the Arabian Peninsula, containing valuable genetic resources for crops and medicines. Its flora is also a mixture of endemic species and elements from Asia, Africa, and the Mediterranean region, in addition to its large number of endemic species [24]. Faifa Mountains are characterized by relatively rich and diverse flora. Approximately 63% of the Jazan flora is in Faifa Mountains [25]. As part of the ethnobotanical culture in the area, the demand for plant-based medicines increases, the natural habitats of Saudi Arabia are under tremendous pressure, and the available data make it difficult to differentiate medicinally important species. Therefore, this study aimed to identify plant species wildly found within Faifa Mountains range in Saudi Arabia, using two barcode loci (the rbcL gene and ITS region) for authentication and surveying.

## 2. Materials and Methods

### 2.1. Study Area 

**Figure 1:**
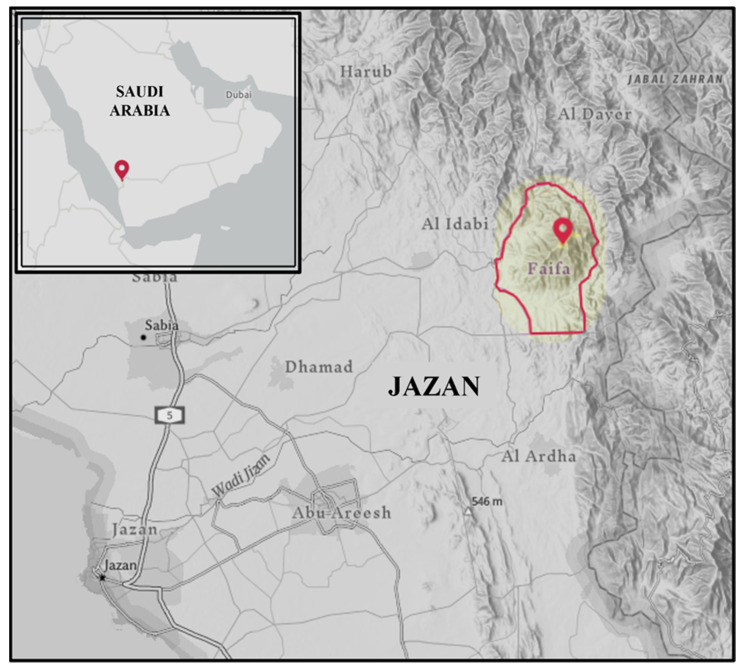
Map of the Kingdom of Saudi Arabia displaying the location of the sampling site (Faifa Mountains).

The study examined the flora inhabit Faifa Mountains (17°15′ N 43°06′ E), a mountain range located in the southwestern region of the Jazan province, Saudi Arabia (17°15′ N 43°06′ E) (Figure 1). Mountains in this region range in elevation from 400 m to about 2000 m.

### 2.2. Sample Collection

Fourteen different species of plants belonging to 12 families were collected from their high-altitude natural habitats during the summer of 2021. A leaf sample was collected from each species (approximately 25 g), and all samples were labeled with a site code and dried immediately with silica gel at room temperature for DNA extraction. Species identification and assignment were independently confirmed prior to the molecular studies and were based on an assessment of morphological descriptors (Tropicos.org).

### 2.3. DNA Extraction, PCR Amplification, and Sequencing

The total genomic DNA of each sample was isolated from ~ 200 mg of dried leaves using the WizPrep™ gDNA Mini Kit (Cell/Tissue; Wizbiosolutions Inc, Gyeonggi-do, Republic of Korea), according to the manufacturer’s instructions, with a final elution volume of 50 mL. The isolated DNA was tested for quality by 1% gel electrophoresis and visualized under UV light using the Ingenius3 Gel documentation system (Syngene, Cambridge, UK). Extracted DNA was stored at −20 °C until required for PCR.

In this study, one plastid barcode region (rbcL gene), and one nuclear ribosomal barcode region, the Internal Transcribed Spacer (ITS) were amplified, identified, and sequenced. The primer pairs for the rbcL gene (rbcLa-F: 5′-TGT CAC CAC AAA CAG ACT AAA GC-3′ and rbcLa-R: 5′-GTA AAA TCA AGT CCA CCR CG-3′), and the ITS region (ITS-U1: 5′-GGA AGK ARA AGT CGT AAC AAG G-3′ and ITS-U4: 5′-RGT TTC TTT TCC TCC GCT TA-3′), were designed by Levin et al. [26] and Cheng et al. [27], respectively. The PCR reaction was performed using One PCR ™ Plus (GenedireX^®®^, Taiwan) master mix in a total volume of 25 μL, containing 12.5 μL of OnePCR™, 1 μL of each primer (forward and reverse, each of 10 μM), and 1 μL of extracted DNA (~100 ng/μL). The optimized PCR profile for both rbcL and ITS comprised of an initial denaturation at 95 °C for 5 min, followed by 35 cycles of 94 °C for 1 min, annealing for rbcL and ITS at 50 °C for 30 s, extension at 72 °C for 90 s, and a final extension segment at 72 °C for 10 min. The amplified PCR products were visualized on 1.5% agarose gels stained with ethidium bromide. Amplicon sizes were confirmed by comparing with the 1Kb DNA ladder (GenedireX^®®^, Taiwan), and successful amplifications were purified by a spin column using EasyPure PCR Purification Kit (TransGen Biotech, Beijing, China) following the manufacturer’s instructions. Purified PCR products were submitted for commercial sequencing in both directions through the Sanger method (Macrogen Inc., Seoul, Republic of Korea).

### 2.4. Sequence Alignment and Data Analysis

After sequencing, the chromatograms obtained were further analyzed using Geneious R10 [28]. To check the quality of each sequence, the peaks corresponding to each nucleotide were examined, and a consensus sequence was produced after trimming the poor-quality DNA sequence ends and aligning forward and reverse sequences. The consensus sequences were identified using the BLAST search tool in the NCBI database, applying default parameters. Each sequence of the rbcL gene and ITS region were aligned separately with the BLAST query results using the MAFFT aligner [29], implemented in Geneious R10. The phylogenies for each gene region were generated using maximum likelihood methods (ML). The ML tree was reconstructed in MEGA X with 1000 bootstrap repeats [30]. Other types of analysis were performed to investigate the influence of the various phylogenetic estimation methods on our results. We carried out Bayesian analysis with the parallel version of MrBayes 3.2 [31], using the HKY85 model implemented in Geneious R10.

## 3. Results

### 3.1. Morphological Observation and Provisional Identification

**Figure 2:**
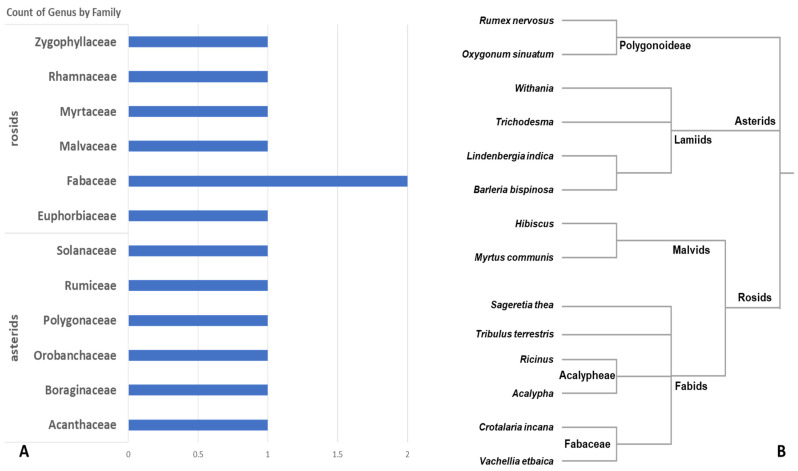
The counts (**A**) and the literature-based phylogeny (**B**) of the fourteen collected plant samples from the Faifa mountains are based on observation and visual inspection.

All the observed plant samples were identified as flowering plants belonging to the class Magnoliopsida (Angiosperms). The 14 plants were found evenly to present two major clades, the Asterids (four species) and the Rosids (eight species), as well as two species of the unclassified order Caryophyllales. In the case of the Asterids, all the samples were identified as Lamiids, where three orders were presented. The order Lamiales was presented by two species of the families, Acanthaceae and Orobanchaceae, namely *Barleria prionitis* and *Lindenbergia siniaca*, respectively. The other orders were uniquely presented by *Trichodesma boissieri* (family Boraginaceae, order Boraginales) and *Withania somnifera* (family Solanaceae, order Solanales; Figure 2).

In the case of the Rosids, two clades were identified, the Malvids presented by the *Hibiscus micranthus* (family Malvaceae, order Malvales), and *Myrtus communis* (family Myrtaceae, order Myrtales). The other clade of the Fabids was presented in four orders, order Zygophyllales of single species *Tribulus terrestris* (family Zygophyllaceae), and order Rosales of single species *Sageretia thea* (family Rhamnaceae), order Malpighiales presented by two species, *Acalypha* sp. and *Ricinus communis* (family Euphorbiaceae), and the order Fabales presented by two species of the family Fabaceae, namely *Crotalaria incana* and *Vachellia tortilis* (Figure 2).

### 3.2. Amplification, Sequencing, and Identification

#### 3.2.1. Chloroplast rbcL Gene

A. BLAST-based identification

The retrieved rbcL sequences ranged between 450 to 454 bp with an average of 452 ± 2 bp; the sequenced quality was Q20 = 99.6%. The rbcL sequence of each sample was used to perform BLAST independently to retrieve top hits available in the database and filtered by > 95% pairwise identity (PI). The BLAST search found that the species (01) matched *Rumex nepalensis* (KX015758; PI = 99.7%), species (02) matched *Oxygonum sinuatum* (KR736460; PI = 100%), species (03) matched *Withania somnifera* (MK142783; PI = 98%), species (04) matched *Trichodesma africanum* (AM234930; PI = 100%), species (05) matched *Lindenbergia* sp. (AJ001768; PI = 97.9%), species (06) matched *Barleria prionitis* (MZ461574; PI = 99.5%), species (07) matched *Hibiscus sabiensis* (MZ461583; PI = 99.8%), species (08) matched *Myrtus communis* (MN662653; PI = 100%), species (09) matched *Sageretia thea* (OL537744; PI = 100%), species (10) matched *Tribulus terrestris* (MN205307; PI = 99.5%), species (11) matched *Ricinus communis* (MT555092; PI = 99.7%), species (13) matched *Crotalaria incana* (KR737341; PI = 100%), and species (14) matched *Vachellia tortilis* (KX015750; PI = 99.6%). In the case of species (03, 09, 13, and 14), additional species were matched at the same PI value. Namely, *Withania coagulans* (NC_047176) matched species (03), *Sageretia paucicostata* (MN722394), and *Sageretia lucida* (MN205213) matched species (09), *Crotalaria* sp. (KR737341) matched species (13), and *Vachellia reficiens* (MK285283) matched species (14) (Table 1).

B. Phylogeny-based identification

**Figure 3:**
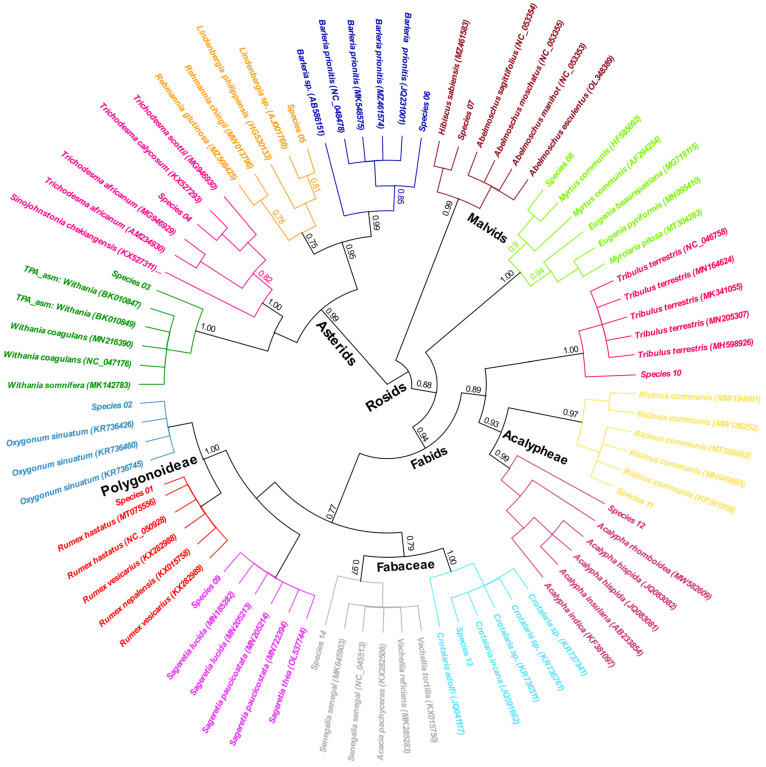
Maximum-likelihood rooted circular phylogenetic tree based on rbcL gene. Each of the studied families is colored differently.

The rbcL sequences, along with the BLAST top 5 hits, were aligned together and trimmed to equal length. The retained total nucleotide alignment was 452 bp, with total identical sites of 334 (73.9%), PI = 91.8%, and Q20 of at least 99.6% of the retained nucleotides. The nucleotide frequencies of non-gaped sites were 26%, 22.2%, 23.7%, and 28.1% for A, C, G, and T, respectively, with GC% = 45.9%. The maximum likelihood tree was constructed based on the aligned sequences and visualized as a rooted circular cladogram (Figure 3). 

The phylogenetic signals were highly in accordance with the registered taxonomical information in the taxonomy database (NCBI), where the two major clades corresponding to the Asterids and the Rosids were distinguished. However, the two species belonging to the unranked Polygonoideae subfamily were grouped with the Fabaceae family and formed part of the fabids, namely (species 01 and 02). The monophyletic observation for species (01) prevented its clear classification at the species level, in contrast to species (02) where the species can confidently be identified as *Oxygonum sinuatum* (bootstrap value = 100). Species (03) was correctly clustered with other members of the genus Withania (family Solanaceae) but not closely related to any specific accession. The same case was observed for species (10, 12, and 14) representing genera Tribulus (family Zygophyllaceae), Acalypha (family Euphorbiaceae), and Senegalia (family Fabaceae), respectively (all bootstrap value > 0.97). Species (04) showed a different clustering than the matched BLAST hit, in which the species clustered with *Trichodesma calycosum* rather than *T. africanum*. The correct taxonomical assignment of the studied species was observed for species (05, 06, 07, and 08), where the studied species were closely or clearly clustered with the matched species at high bootstrap support (>0.80). The monophyletic case was also found for species (09) that impeded the correct species assignment of this sample to a certain species from the genus Sageretia (family Rhamnaceae; Figure 3). The species (13) was clustered with three unidentified species and one known species of the genus Crotalaria; thus, the whole clade, including the sample understudy, was assigned as *C. incana* (family Fabaceae).

#### 3.2.2. Nuclear ITS Region

A. BLAST-based identification

The retrieved ITS sequences ranged between 518 to 684 bp with an average of 601 ± 83 bp; the sequenced quality was Q20 = 97.5%. The ITS sequence of each sample was used to perform BLAST independently to retrieve top hits for each plant sample. Limited by the database and filtered by > 95% pairwise identity (PI), the species (01) matched *Rumex nepalensis* (AF338219; PI = 100%), species (02) matched *Oxygonum sinuatum* (KR537784; PI = 99.5%), species (03) matched *Withania somnifera* (KY675295; PI = 99.4%), species (04) matched *Trichodesma boissieri* (KP027117; PI = 97.2%), species (05) matched *Lindenbergia siniaca* (KY513938; PI = 94.5%), species (06) matched *Barleria prionitis* (MK066159; PI = 100%), species (07) matched *Hibiscus micranthus* (KF850572; PI = 98.9%), species (08) matched *Myrtus communis* (JQ740194; PI = 100%), species (09) matched *Sageretia thea* (MK000448; PI = 100%), species (10) matched *Tribulus terrestris* (KF850577; PI = 99.6%), species (11) matched *Ricinus communis* (KF850582; PI = 99.8%), species (13) matched *Crotalaria incana* (KP698656; PI = 100%), species (14) matched *Vachellia tortilis* (MH547553; PI = 100%), and species (12) matched *Acalypha* sp. (MN257839; PI = 99.6%). In the case of species (05 and 09), additional species were matched at the same PI value. Namely, *Lindenbergia indica* (KF850597) and *Sageretia omeiensis* (MK000463) matched species (05) and (09), respectively (Table 2).

B. Phylogeny-Based Identification

The ITS sequences, along with the BLAST top 5 hits, were aligned together and trimmed to equal length. The retained total nucleotide alignment was 762 bp, with total identical sites of 187 (24.5%), PI = 54.5%, and Q20 of at least 95.7% of the retained nucleotides. The nucleotide frequencies of non-gaped sites were 19%, 29.3%, 31.3%, and 20.4% for A, C, G, and T, respectively, with GC% = 60.6%, while the gaps were 22.5% of the total alignment. 

**Figure 4:**
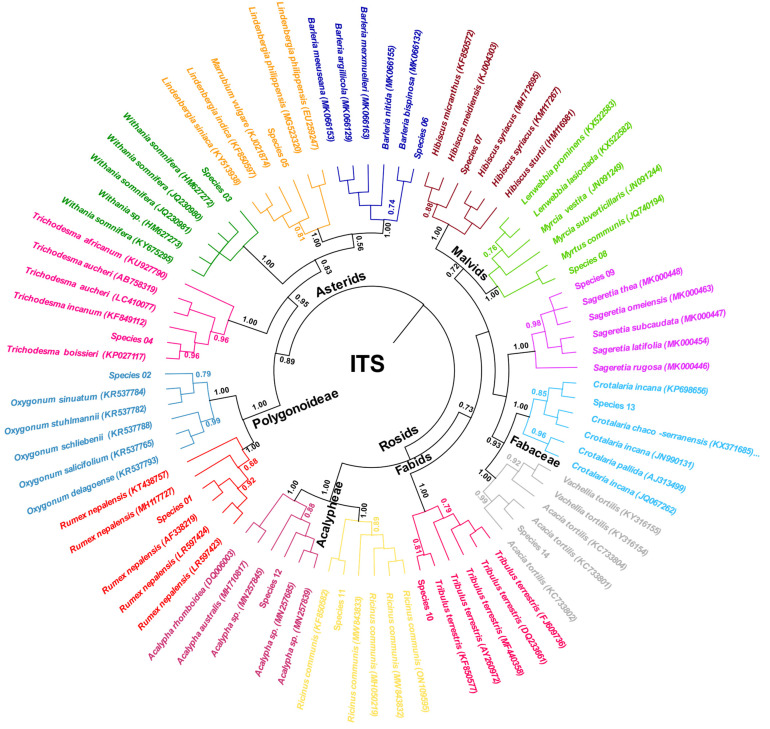
Maximum-likelihood rooted circular phylogenetic tree based on nuclear ITS region.

Based on the aligned sequences, the maximum likelihood tree was constructed and visualized as a rooted circular cladogram (Figure 4). The phylogenetic clustering is in accordance with the published taxonomical information only at the high ranks. In detail, the two major clades corresponding to the Asterids and the Rosids were defined, as well as the unranked Polygonoideae subfamily. However, all species of the same family were clustered together correctly, but the families were not grouped in accordance with known taxonomical information. Species (01 and 02) were correctly clustered with other members of the Polygonoideae subfamily and closely related to each other and were defined as the BLAST results. Equally, the correct taxonomical assignment at the family level of the studied species was observed for species (03, 04, 05, and 06) presenting the Asterids, and clustered with *Withania somnifera*, *Trichodesma boissieri*, Lindenbergia species, and *Barleria bispinosa*, respectively. Species (07 and 08) were assigned correctly to the Malvids, but species (07) was not clustered to a single species of the genus Hibiscus, in contrast to species (08), which was clustered clearly with *Myrtus communis*. Species (09) showed a monophyletic status with two Sageretia species; thus, its identity remains uncertain. Species (10) was clustered incorrectly superior to malvids; it represents the fabids and is correctly distinguished as *Tribulus terrestris* (bootstrap values > 0.80). The family Euphorbiaceae was represented by two genera, Acalypha sp. (species 12) and *Ricinus communis* (species 11). The species (13 and 14) of the Fabaceae family were clearly identified with high bootstrap values (>0.80) as *Crotalaria incana* and *Acacia tortilis* (syn. *Vachellia tortilis*; Figure 4).

### 3.3. Integrative Comparative Analysis

The comparison between the morphological inspection versus the DNA barcoding identification showed agreements as well as disagreements. Based on the BLAST results, the molecular identification using both molecular loci agreed with the morphological inspection for species 02, 08, 09, 10, and 13, as *Oxygonum sinuatum*, *Myrtus communis*, *Sageretia thea*, *Tribulus terrestris*, and *Crotalaria incana*, respectively. Species 01, 03, 06, 11, and 14 were equally identified between rbcL and ITS in most cases (except for species 14) but were not equal to the morphological inspection. Those species were identified as *Rumex nepalensis*, *Withania somnifera*, *Barleria prionitis*, *Ricinus communis*, and *Vachellia tortilis*, respectively. 

Total disagreement between the morphological inspection, ITS, and rbcL was found at the species level for species 04, 05, 07, and 12. In the case of species 04, it was confusing to have a clear match with three different species for each of the markers, namely *Trichodesma calycaroum* (rbcL) and *Trichodesma boissieri* (ITS). However, the rbcL BLAST-based identification was matching with *Trichodesma africanum*. Species 05 and 12 were both of unknown species of the genus Lindenbergia and Acalypha, where none of the two markers matched a species with certainty. The rbcL phylogenetic analysis showed enough genetic variation to delimit species by paraphyletic clustering for species 07 in contrast to the ITS monophyletic clustering for this species, identified as *Hibiscus sabiensis* (Table 3).

## 4. Discussion

The drug’s efficacy decreases if it is adulterated, and in some cases, it can be lethal if it is substituted with toxic adulterants [16]. The adulteration of herbal materials usually occurs due to materials not having readily distinguishable morphological characters or the substitution of economically valuable materials with inexpensive herbs [16]. Hence, correctly identifying medicinal plants using genetics may enhance the quick and precise identification of species of economic interest. 

To standardize the international use of DNA barcodes, the scientific community has made considerable efforts to search for suitable DNA regions to barcode every species [32]. After an extensive inventory of gene regions in the mitochondrial, plastid, and nuclear genomes, the nuclear ITS region and the chloroplast genes rbcL and/or matK have generally been agreed upon as the standard DNA barcodes of choice and were recommended by the Consortium for the Barcode of Life (CBOL) as a standard two-locus barcode for global plant databases because of their species discrimination ability together [33]. Indeed, in our analysis, by comparing ITS and rbcL phylogenetic analysis, we found that the ITS tree was better at defining taxonomy at the family or lower levels in contrast to the rbcL, which was able to define higher taxonomical ranks. Although ITS was more efficient in differentiating species, using it solely will not be recommended due to the variation within species [22,34,35,36]. Combining both regions guided by morphological inspection helped identify the wild species. In the current analysis, we identified 10 out of the 14 species, five of which were identified as morphologically inspected, in contrast to three species where the morphological inspection was indifferent. In one case, species (4) differed by morphological inspection, and both barcodes were incongruent. Two species were morphologically inspected as *Lindenbergia siniaca* and *Acalypha fruticose*, but the barcodes were not successfully identified based on the NCBI database. Database and sequence search strategies are the essential limits to the success of the barcoding technique for species identification [37].

The wild plants are usually enlisted as medicinal and ethnobotanical “folk medicine” plants. Our study found a list of certain species that can be identified as medicinal plants. However, as previously mentioned, the identification of medicinal plants has had a long history, and the correct identification of these plants is a prerequisite for their safe application. For example, a plant of medicinal value (contains flavonoids, anthraquinones, and gallic acid) has been reported from the sampling region in Saudi Arabia as *Rumex nervosus* [38], a sample that we equally have inspected. However, the DNA barcoding using both markers confirmed that the species is *Rumex nepalensis*, which can raise doubts to its safe application without incorporating an effective identification tool as the DNA barcoding. Similarly, another sample was identified by DNA as *Barleria prionitis*, even thought it was morphologically identified as *Barleria bispinosa*, a native species to the Arabian peninsula [39], which again raise doubts around the common wild plants identified in the region, as well as the safe application of this species, as several species of Barleria are known for their medicinal or ornamental values, but not all [40]. *Vachellia* (*syn. Acacia*) *etbaica* is a wooden wild plant growing in the desert of Egypt and proximate deserts around [41], a plant we found in our study, but was proven by DNA to be a proximate species *Vachellia tortilis*, a medicinal tree that has edible gum and can be used as Arabic Gum [42]. An interesting finding is the presence of *Trichodesma boissieri*, a plant that was only identified by the ITS phylogenetics, a plant that has been reported from the northern parts of the Arabian Peninsula [39] but has never been reported that far into the south. The climate change effect on the sampling area may contribute to the vegetation diversity detected in the Faifa mountains. Climate change affects species distributions through changes in plant growth and reproduction; it can act directly (e.g., drought, wind) and indirectly (e.g., temperature and disease outbreaks) [43]. 

Based on our findings, we recommend an in situ conservation plan for those wild medicinal plant species; it has a valuable role to play in maintaining genetic resources for folk medicinal plants and allowing for the continued adaptation and evolution of migrated plant genotypes.

## Figures and Tables

**Table 1:** Blast results of the rbcL sequences of 14 medicinal species of Faifa mountains.

Sample	Species	PI%	Accession No.
Species_01	*Rumex nepalensis*	99.70%	KX015758
Species_02	*Oxygonum sinuatum*	100%	KR736460
Species_03	*Withania somnifera*	98.00%	MK142783
	*Withania coagulans*		NC_047176
Species_04	*Trichodesma africanum*	100%	AM234930
Species_05	*Lindenbergia* sp.	97.90%	AJ001768
Species_06	*Barleria prionitis*	99.50%	MZ461574
Species_07	*Hibiscus sabiensis*	99.80%	MZ461583
Species_08	*Myrtus communis*	100%	MN662653
Species_09	*Sageretia thea*	100%	OL537744
	*Sageretia paucicostata*		MN722394
	*Sageretia lucida*		MN205213
Species_10	*Tribulus terrestris*	99.50%	MN205307
Species_11	*Ricinus communis*	99.70%	MT555092
Species_12	*Acalypha indica*	99%	KF381097
Species_13	*Crotalaria* sp.	100%	KR737341
	*Crotalaria incana*		JQ591662
Species_14	*Vachellia tortilis*	99.60%	KX015750
	*Vachellia reficiens*		MK285283

**Table 2:** Blast results of the ITS sequences of 12 rare species of Faifa mountains.

Sample	Species	PI%	Accession No.
Species_01	*Rumex nepalensis*	100%	AF338219
Species_02	*Oxygonum sinuatum*	99.50%	KR537784
Species_03	*Withania somnifera*	99.40%	KY675295
Species_04	*Trichodesma boissieri*	97.20%	KP027117
Species_05	*Lindenbergia siniaca*	94.50%	KY513938
	*Lindenbergia indica*		KF850597
Species_06	*Barleria prionitis*	100%	MK066159
Species_07	*Hibiscus micranthus*	98.90%	KF850572
Species_08	*Myrtus communis*	100%	JQ740194
Species_09	*Sageretia thea*	100%	MK000448
	*Sageretia omeiensis*		MK000463
Species_10	*Tribulus terrestris*	99.60%	KF850577
Species_11	*Ricinus communis*	99.80%	KF850582
Species_12	*Acalypha* sp.	99.60%	MN257839
Species_13	*Crotalaria incana*	100%	KP698656
Species_14	*Vachellia tortilis*	100%	MH547553

**Table 3:** Comparative identification summary based on the morphological inspection, rbcL, and ITS DNA barcodes of 14 rare plants of Faifa mountains. Uncertain species were written underlined, while equal species identifications were written in bold.

Sample	Morphology	rbcL	ITS
Species_01	*Rumex nervous*	***Rumex nepalensis***	***Rumex nepalensis***
Species_02	***Oxygonum sinuatum***	***Oxygonum sinuatum***	***Oxygonum sinuatum***
Species_03	*Withania* sp.	***Withania somnifera***	***Withania somnifera***
Species_04	*Trichodesma* sp.	*Trichodesma calycosum*	*Trichodesma boissieri*
Species_05	*Lindenbergia siniaca*	*Lindenbergia* sp.	*Lindenbergia* sp.
Species_06	*Barleria bispinosa*	***Barleria prionitis***	***Barleria prionitis***
Species_07	*Hibiscus* sp.	*Hibiscus sabiensis*	*Hibiscus* sp.
Species_08	***Myrtus communis***	***Myrtus communis***	***Myrtus communis***
Species_09	***Sageretia thea***	***Sageretia thea***	***Sageretia thea***
		*Sageretia paucicostata*	*Sageretia omeiensis*
		*Sageretia lucida*	
Species_10	***Tribulus terrestris***	***Tribulus terrestris***	***Tribulus terrestris***
Species_11	*Ricinus* sp.	***Ricinus communis***	***Ricinus communis***
Species_12	*Acalypha fruticosa*	*Acalypha* sp.	*Acalypha* sp.
Species_13	***Crotalaria incana***	***Crotalaria incana***	***Crotalaria incana***
Species_14	*Vachellia etbaica*	***Vachellia tortilis***	***Vachellia tortilis***
		*Vachellia reficiens*	

## Data Availability

Authors declare that all the data supporting the findings of this study are available under the GenBank (NCBI).
